# Physiological Roles of Serotonin in Bivalves: Possible Interference by Environmental Chemicals Resulting in Neuroendocrine Disruption

**DOI:** 10.3389/fendo.2022.792589

**Published:** 2022-02-25

**Authors:** Laura Canesi, Angelica Miglioli, Teresa Balbi, Elena Fabbri

**Affiliations:** ^1^ Environmental Physiology Laboratory, Department of Earth, Environment and Life Sciences, University of Genoa, Genoa, Italy; ^2^ Laboratoire de Biologie du Developpement de Villefranche-sur-mer, Institut de la mer, Sorbonne Université, CNRS, Villefranche-sur-mer, France; ^3^ Department of Biological, Geological, and Environmental Sciences, University of Bologna, Ravenna, Italy

**Keywords:** contaminants of emerging concern, neuroendocrine system, bivalve molluscs, serotonin signaling, neuroendocrine disrupting chemicals, pharmaceuticals, larvae

## Abstract

Contaminants of Emerging Concerns (CECs) are defined as chemicals not commonly monitored in aquatic ecosystems, but with the potential to cause adverse effects on biota. CECs include Endocrine Disrupting Chemicals (EDCs) and Neuro-Endocrine disruptors (NEDs) of vertebrates. However, most invertebrates only rely on neuroendocrine systems to maintain homeostatic processes. Although conserved neuroendocrine components have been characterized in ecologically relevant groups, limited knowledge on invertebrate neuroendocrinology makes it difficult to define EDCs and NEDs in most species. The monoamine serotonin (5-hydroxytryptamine, 5-HT) acts both as a neurotransmitter and as a peripheral hormone in mammals. In molluscs, 5-HT is involved in multiple physiological roles and molecular components of the serotonergic system have been identified. This review is focused on the effects of CECs on the serotonergic system of bivalve molluscs. Bivalves are widespread in all aquatic environments, estuarine and coastal areas in particular, where they are exposed to a variety of chemicals. In bivalves, 5-HT is involved in gametogenesis and spawning, oocyte maturation and sperm motility, regulates heart function, gill ciliary beating, mantle/siphon function, the ‘‘catch’’ state of smooth muscle and immune responses. Components of 5-HT transduction (receptors and signaling pathways) are being identified in several bivalve species. Different CECs have been shown to affect bivalve serotonergic system. This particularly applies to antidepressants, among the most commonly detected human pharmaceuticals in the aquatic environment. In particular, selective serotonin reuptake inhibitors (SSRIs) are frequently detected in seawater and in bivalve tissues. Information available on the effects and mechanisms of action of SSRIs on the serotonergic system of adult bivalves is summarized. Data are also reported on the effects of CECs on development of neuroendocrine pathways of early larval stages, in particular on the effects of model EDCs in the marine mussel *Mytilus galloprovincialis*. Overall, available data point at the serotonergic system as a sensitive target for neuroendocrine disruption in bivalves. The results contribute drawing Adverse Outcome Pathways (AOPs) for model EDCs and SSRIs in larvae and adults. However, basic research on neuroendocrine signaling is still needed to evaluate the potential impact of neuroendocrine disruptors in key invertebrate groups of aquatic ecosystems.

## Introduction

Contaminants of emerging concern (CECs) are defined as naturally occurring or manufactured substances which have recently been discovered or present for a long time but only recently recognized as widely occurring and potentially dangerous (1). In general, they are not yet subjected to regulatory criteria or norms for the protection of human health or the environment. Examples of CECs include endocrine disrupting chemicals (EDCs), plasticizers, pharmaceutical and personal care products, disinfection by-products, various fluorinated compounds, as well as their degradation products, nanomaterials, and also some legacy contaminants such as arsenic, lead etc. (1). Although CECs are virtually found in any environmental compartment, aquatic in particular, “exposure to low concentrations of CECs may not cause overt toxicity, but rather subtle changes in the health and physiology of the organisms that have the potential to cause adverse ecological outcomes in terms of population levels and biodiversity” (2).

With regards to EDCs, in line with the most recent consensus publications, interference with the hormonal system is the *conditio sine qua non* for the identification of ‘key characteristics’ of EDCs as such (3). However, this approach is essentially based on knowledge of endocrine systems of vertebrates, which makes it particularly difficult to apply consistently the consensus guidelines on EDCs to invertebrates, which represent the majority of species in the animal kingdom. This is because information on invertebrate hormones and endocrine systems is only partly available for arthropods, and extremely limited and scattered for other phyla and most species. However, several invertebrates represent key components of aquatic ecosystems, where they are potentially exposed to a variety of potential EDCs (4, 5). Substantially, the lack of detailed comprehension of the endocrine signaling pathways in invertebrates represents the main gap of knowledge on endocrine disruption in ecologically relevant groups (4, 6, 7).

In the last few years, it has become clear that a wide variety of CECs have specific effects on neuroendocrine systems of vertebrates, acting as Neuro Endocrine disruptors (NEDs) (8–10). Actually, the majority of EDCs have already been found to be also NEDs in vertebrate models (11).

However, most invertebrates are not endowed with a canonical endocrine system, but only with a neuroendocrine system that exerts a variety of complex regulatory and developmental functions through the secretion of neuropeptides and neurotransmitters (12, 13). A neurosecretory/neuroendocrine system appeared very early in metazoan history, so that all invertebrates possess neuroendocrine cells releasing signaling molecules in the hemolymph or coelomic fluid; moreover, in many invertebrates, such as polychaetes and molluscs, several neurons, although not organized in a gland-like structure, were proved to be neurosecretory cells (14, 15). Accordingly, it could be argued that endocrine disruption in invertebrates is mainly neuroendocrine. However, the scarce knowledge on neuroendocrinology of most invertebrate species makes it difficult to define EDCs/NEDs in these organisms. An alternative approach considers the investigation of shared molecular pathways in vertebrates and invertebrates that are susceptible to EDCs, regardless their possible involvement in the neuroendocrine system of invertebrates (7).

In this work, we will focus on one of the most conserved neuroendocrine pathway, i.e. the serotonin (5-hydroxytryptamine, 5-HT) signalling, as a potential target for EDCs and NEDs in bivalve molluscs, a large invertebrate group widespread in all aquatic environments, estuarine and coastal areas in particular, where they are subjected to contamination by a variety of chemicals.

## Pleiotropic Functions of Serotonin in Bivalves

In the early sixties, 5-HT was identified in the nervous system of bivalves at levels that were much higher than those of other invertebrates and vertebrates (16). Serotonin is a main actor in the neuroendocrine-immune regulation in marine bivalves, and it participates efficient responses to environmental stressors, including osmotic or temperature changes, acidification and pollution [see for review ref. (17)]. However, many other physiological functions of 5-HT have long been reported in bivalves and are shortly described below.

*Gametogenesis and spawning*: serotonin is physiologically produced in the nervous system, and delivered through serotoninergic fibers identified in the mantle/gonads of several bivalve species (17). Accordingly, different reproductive events are regulated by 5-HT in bivalves. Serotonin modulates the availability of energetic substrates supporting gametogenesis. Glycogen, that represents the main source of energy, is accumulated during the nonbreeding season in the storage cells of the mantle, then used as a fuel for gametogenesis up to the time of spawning (18). In *Mytilus galloprovincialis*, 5-HT-induced increase in cyclic adenosine monophosphate (cAMP) levels plays a relevant role in the regulation of stored glycogen breakdown during the annual gametogenic cycle (18). In several species of bivalves, injection of 5-HT was shown to induce spawning within minutes (19), and it is now acknowledged that 5-HT is the most potent inducer of spawning in bivalves (20). Exogenous 5-HT acts both on ovaries and testis in a dose-dependent manner, with females appearing less sensitive than males (20). In fingernail clams (*Sphaerium* spp), ovoviviparous freshwater bivalves, exogenous 5-HT potently induced parturition (21).

Serotonin also shows direct actions on bivalve gametes: it triggers meiosis re-initiation of the prophase-arrested cells in ovaries of *Hiatella flaccida* and other bivalves (22), thus ensuring germinal vesicle breakdown and transition toward the metaphase, the physiological stage at which fertilization occurs. Exposure of prophase-I oocytes to 5-HT caused an increase in intracellular [Ca^2+^], both from internal stores and external influx, and a gradual rise in intracellular pH: these events are considered responsible for the release from prophase-I arrest (22).

Bivalve sperm motility is induced after release in seawater. Stimulation of sperm motility is due to an increase in intracellular pH mediated by a membrane Na^+^/H^+^ exchanger that, probably through modulation of the activity of a Na^+^/Ca^2+^ exchanger, leads to an increase in intracellular [Ca^2+^]. This in turn triggers Ca^2+^/calmodulin-dependent flagellar beating (23). In various bivalve species it has been shown that 5-HT is capable to further enhance sperm motility, and pharmacological approaches suggested the involvement of different 5-HT receptor isoforms. The mechanism is not clear yet; however, a cAMP-protein kinase A (PKA) dependent phosphorylation in the dynein α chain of the flagellum, possibly modulating its flexibility, is suggested (23).

*Heart function*: in the ventricle of the clam *Mercenaria mercenaria* a positive correlation between intracellular cAMP levels and the increase in contractility was observed after treatment with 5-HT (24). When 5-HT and other monoamines were tested in the isolated heart of the marine bivalve *Meretrix lusoria*, and only 5-HT produced positive chronotropic and inotropic effects at physiological concentrations (0.01 nM) (25). It was concluded that 5-HT is the excitatory agent that regulates the cardiac performance in this as in other marine bivalves.

*Gill ciliary beating*: in the gills of most bivalves the water currents that provide for respiration and feeding are mainly created by lateral cilia, with a smaller contribution by abfrontal cilia (26). In the gills of *Crassostrea virginica* 5-HT activated the movements of lateral cilia at a frequency proportional to the neuromodulator concentration, whereas dopamine (DA) had an inhibitory effect (27). In contrast, normal beating of laterofrontal cilia was not controlled by 5-HT. However, when the organism flushes the mantle cavity without feeding, the beating of laterofrontal cilia is arrested by high concentrations of 5-HT released from the serotoninergic fibers (27). In isolated gills from *M. mercenaria*, a biphasic effect of 5-HT was observed on ciliary movement during clearance of colloidal graphite, with increases at 1-10 µM 5-HT and decreases at higher concentrations (28).

*Mantle/siphon function:* exogenous 5-HT showed similar concentration-dependent effects also in the mantle/siphon region of the zebra mussel *Dreissena polimorpha*, that is innervated by serotonergic fibers (29). Serotonin induced the contractile response at 10 µM and relaxation at 1 µM. Higher concentrations (1mM) induced siphon opening. Although the mechanisms involved are unknown, they are probably different from those involved in 5-HT-induced spawning, since none of the 5-HTR antagonists that inhibit mussel spawning had any effect on the siphon response to serotonin (29).

*‘Catch’ Muscle*: in bivalves, smooth muscles, such as the adductor and the anterior byssus retractor muscle, can be locked in the contracted state, i.e. the “catch’’ state, a crucial function that keep their shells firmly closed during the periods of aerial exposure (30). Catch occurs following the initial activation of the muscle, and it is characterized by prolonged force maintenance in the face of a low [Ca^2+^]_i_, high instantaneous stiffness, a very slow cross-bridge cycling rate, and low ATP usage (30). In the past, a considerable body of experimental data highlighted the role of 5-HT and cAMP in the control of catch (32 and refs. therein). Tension is maintained until serotoninergic fibers release 5-HT, which stimulates the AC/cAMP/PKA system. PKA is then responsible for muscle rapid relaxation through phosphorylation of twitchin, a myosin binding protein (31).

*Immunity*: serotonin is known to play a crucial role in immunomodulation in vertebrates, including humans, by regulating the synthesis of interleukin-6 and tumor necrosis factor (TNF) mRNA, promoting the expression and activity of superoxide dismutase (SOD) (32), and reducing apoptosis (33). Serotonin is of vital importance in neuroendocrine-immune regulation also in bivalves, to protect the host from pathogen infection. A 5-HT1 receptor was identified from *C. gigas* (Cg5-HTR-1) showing high expression in digestive gland and hemocytes (34). In oyster hemocytes, lipopolysaccharide (LPS) induced the over-expression of Cg5-HTR-1 mRNA, while pre-treatment with the receptor blocker methiothepin lead to down-regulation of TNFα mRNA and SOD activity, and to increased apoptosis. The authors concluded that Cg5-HTR-1 plays a crucial role in modulating the immune response to pathogens or in maintaining immune homeostasis (34). Transfection of the Cg5-HTR-1 in HEK293T cells demonstrated that treatment with 5-HT caused inhibition of Adenylate cyclase (AC) and reduction in cAMP levels (34).

## Serotonin Signalling

Serotonin receptors (5-HTRs) have been identified in several invertebrate species from diverse phyla, and their molecular classification and pharmacology has been recently reviewed (20, 24, 35). Although 5-HTRs can be grouped by their sequence analysis and conserved primary transduction mechanisms in both vertebrates and invertebrates, the pharmacological profiles differ between invertebrate and mammalian receptors. A few ligands display specificity for different receptors within a single species; however, none acts with high specificity in receptors across different species (35). Moreover, invertebrate receptors are from multiple classes and distantly related phyla, and orthologous relationships among receptor families may occur within, but not necessarily across phyla. Hence, along with the expansion of knowledge of different invertebrate receptors, the development of a precise nomenclature presents significant challenges. Taking into account this complexity, Tierney proposed a classification of invertebrate 5-HTRs with known functional properties (35).

Early molecular studies reported the presence of 5-HTR genes in invertebrates orthologous to mammalian receptors 5-HT1, 5-HT2 and 5-HT7, as determined by similarities in sequence and transduction mechanisms (36). The classification became more complex in the following years and needed clarifications, that are illustrated by Tierney (35).

In molluscs, the 2^nd^ largest invertebrate phylum, different 5-HTRs have been identified. 5-HT1 receptor sequences were detected and named 5-HT 1Lym (from *Limnaea stagnalis*), 5-HT1aApca and 5HT1bApca (from *Aplysia californica*) 5-HT1Heltr (from *Helisoma trivolvis*) and 5-HT1Hal (from *Haliotis asinina*) (35). Only two sequences were reported for Bivalvia, 5-HT1Pat and 5-HT1Pin, from *Patinopecten yessoensis* (37) and *Pinctada fucata* (38), respectively.

The assembly of a 1.28-Gb reference genome of the mediterranean mussel *M. galloprovincialis* has been recently released (39). Due to the lack of information on most components of neuroendocrine signaling in this species, including serotonin and other monoamines, we first made a general search in *M. galloprovincialis* genome for orthologous sequences corresponding to the molecular elements of the three families of monoamines (indolamines, imidazolamines, catecholamines): synthesis and degradation, transport, receptors, and neurotransmitters. The results, reported in **Table 1**, underline the presence of most, if not all, conserved components of monoamine pathways in *M. galloprovincialis*. With regards to components of the serotonergic system, sequences homologous to those of their vertebrate counterparts were identified for enzymes of 5-HT synthesis (tryptophan hydroxylase and 5-tryptophan decarboxylase) and degradation (monoamine oxidase and aldehyde dehydrogenase); transport, serotonin reuptake transporters (SERT) (5-HTT) and vesicular monoamine transporter (VMAT) (SLC18A); 5-HTR isoforms, in particular 5-HTR1 A-F, 5-HTR. Components of metabolism, transport and receptors for other monoamines, i.e. dopamine and noradrenaline, but not for adrenaline, were also identified. This will provide basal information on the potential targets for NED action in *Mytilus*.

**Table 1:** Molecular elements of the three families of monoamine neurotransmitters: enzymes involved in synthesis and degradation, enzymes, transporters, receptors.

	NEUROTRANSMITTERS	SYNTHESIS ENZYMES	DEGRADATION ENZYMES	TRANSPORTERS	RECEPTORS
INDOLAMINES	SEROTONIN (5-HT)	Tryptophan hydroxylase and 5-tryptophan decarboxylase	Monoamine oxidase and Aldehyde dehydrogenase	SERT (5-HTT) and VMAT (SLC18A)	Serotonin receptors: 5-HTR1 A-F, 5-HTR2 A-C, 5-HTR 3-7
	MELATONIN (MT)	Serotonin N-acetyltransferase and Hydroxyindole O-methyltransferase	Proteases	//	Melatonin receptors: MR 1-2
IMIDAZOLAMINES	HISTAMINE	L-histidine decarboxylase	Monoamine oxidase and Histamine-N-methyltransferase	VMAT (SLC18A)	Histamine receptors: RH 1-4, HGCL
CATECHOLAMINES	DOPAMINE (DA)	Tyrosine hydroxylase and Dopamine decarboxylase	Monoamine oxidase and Catecol-O-methyltransferase	Dopamine transporter (SLC6A3) and VMAT (SLC18A)	Dopamine receptors: DR1 (D1, D5), DR2 (D2-4)
	NORADRENALINE	Dopamine β-hydroxylase	Monoamine oxidase and Catecol-O-methyltransferase	Dopamine transporter (SLC6A2) and VMAT (SLC18A)	Adrenergic receptors: α1,2 and β1
	ADRENALINE (AD)	Phenylethanolamine-N-methyltransferase	Monoamine oxidase and Catecol-O-methyltransferase	VMAT (SLC18A)	Adrenergic receptors: β2,3

The elements for which orthologous sequences were identified in the genome of the mediterranean mussel *M. galloprovincialis* (39) are reported in red.

**Figure 1:**
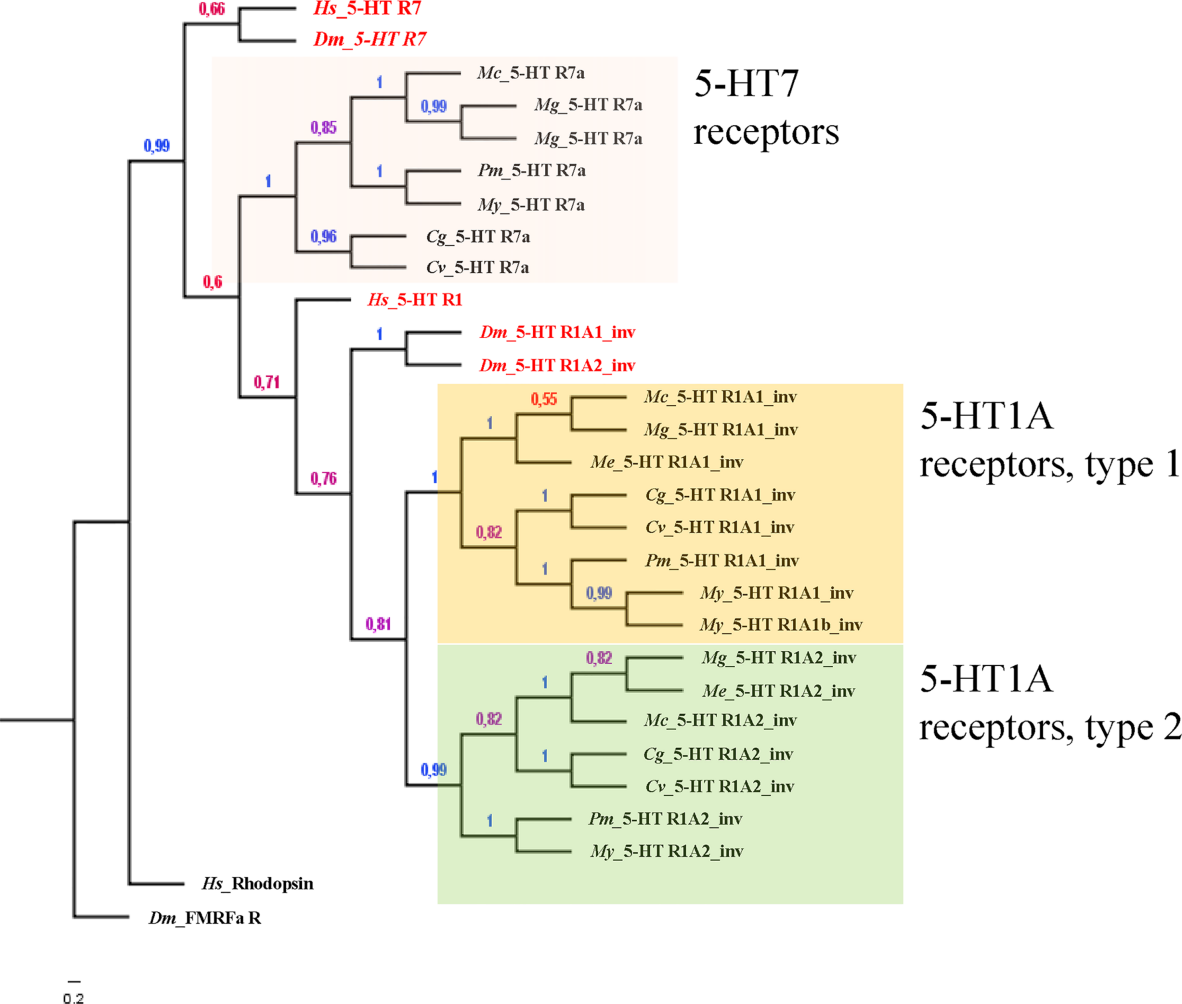
Bayesian phylogeny of 5-HTR bivalve species with respect to the human and fruit fly orthologous sequences. Alignments were performed using Muscle (v.3.5) by using the whole protein sequence. The phylogenetic tree was constructed with MrBayes (v.3.2.3) with default parameters except: generation number = 100.000, rate matrix for aa = fixed (dayhoff), sampling set= 10, and burnin to 25. *D. melanogaster* FMRFamide receptor and *H. sapiens* Rhodopsin were employed as outgroup sequences to root the tree as previously described (40). Numbers at branches represent the posterior probabilities. The distinct groups of receptors are highlighted in different colors.

Alavi et al. (20) also reported the phylogenetic analysis of the 5-HTRs known from invertebrates and from invertebrates and vertebrates, including the three sequences at the time available for 2 bivalve species (namely *Patinopecten yessoensis*, py5-HT and *Pinctada fucata*, pf5-HT). Recent advances in identification of gene sequences allow for an updated list of 5-HTRs of several species of bivalves on a molecular basis (see **Table S1** and **Figure 1**). A search for 5-HTRs in available bivalve genomes coupled with the identification of the conserved functional domain of the corresponding proteins in humans and in the model invertebrate *Drosophila melanogaster* (extracted from the sequences available in the UniprotKB database), reveals that bivalve molluscs possess three 5-HTRs. One has the typical domain of the 5-HT receptor 7 (7tmA_5-HT7) and two show the conserved domain of the invertebrate 5-HT1A receptor (7tmA_5-HT1A_invertebrates) (**Table S1**). The phylogenetic analysis confirms the outcome of the domain search, as bivalve 5-HTRs clustered with the reference amino acid sequences of both *H. sapiens* and *D. melanogaster* 5-HT7 and 5-HT1 (**Figure 1**).

With regards to the pharmacological classification of 5-HTRs, it has been clear for many years that responses to serotonergic drugs differ between invertebrate and vertebrate (mammalian) receptors (36). For a detailed comparison of the pharmacology of 5-HTRs in bivalve molluscs with other vertebrates see (20). How pharmacology varies within and between invertebrate phyla is a challenging question, yet. Some non-specific agonists (5-methoxytryptamine, 5-carboxamidotryptamine, 2-methyl-5-HT) and non-specific antagonists (methiothepin and mianserin) have been used to help understanding invertebrate 5-HTR classification; however, the experimental results were somehow confounding (35). Only few data are available for bivalves. In the gills of *Tapes philippinarum*, 5-HTRs showed high affinity for ligands specific to the mammalian receptors 5-HT1 or 5-HT7; however, on the basis of the rank order of potency butaclamol > dihydroergocryptine > methysergide > prazosin > yohimbine they could not be classified as 5-HT1 or 5-HT7 (41). The pharmacological approach identified a receptor population of a 5-HT1/5-HTR2 mixed profile (41). In the fingernail clams *Spherium* spp. the 5-HTR mediating parturition (possibly located on either gonads or gametes) was pharmacologically characterized (21). The receptor displayed a mixed 5-HTR1/5-HTR2 pharmacological profile, differently from all 5-HTRs described in vertebrates. Such mixed profile seems to be common in bivalves, as it was shown in a number of other representative species (21). At present, no selective ligands for any invertebrate 5-HT receptor subtypes have been identified (35).

In contrast to the variability of pharmacological profiles, transduction mechanisms associated with 5-HTR1, 5-HTR2 and 5-HTR7 appear to be well conserved across invertebrate phyla and between invertebrates and mammals. The receptor families can be grouped by their primary transduction mechanism (42). Receptors 5-HTR1 and 5-HTR5 couple preferentially to Gi/o proteins, leading to the inhibition of AC activity and decreased production of cAMP. In contrast, 5-HTR4, 5-HTR6 and 5-HTR7 couple preferentially to Gs proteins, leading to the activation of AC and increased production of cAMP; 5-HTR2 couple to Gq proteins, and lead to the activation of phospholipase C (PLC) and subsequent increase in IP_3_ and Ca^2+^ intracellular levels (35). The 5-HTR3 is the only member of the family not coupled with G proteins, and acts as a non-selective cation channel (35). As described above, studies on different functional roles of 5-HT in different bivalve species pointed at cAMP/PKA as the main signal transduction pathway activated by serotonin in different tissues (mantle, heart, gills, catch muscle), indicating a Gs-mediated mechanism. In contrast, studies on serotonin signaling in hemocytes of *M. galloprovincialis* provided evidence for the occurrence of 5-HT receptors coupled to Gi/o proteins (43). *In vitro* exposure to 5-HT induced a decrease in cAMP levels, and a significant reduction in cAMP-dependent PKA phosphorylation was observed consequently. The reduction in cAMP levels and PKA phosphorylation are consistent with the occupation of 5-HTR1 by serotonin. In the same experimental setup, Franzellitti et al. (43) also demonstrated that serotonin reduced the expression of the ABCB1 gene product, which encodes the P-glycoprotein membrane transporter. The expression of ABCB1 gene product is under cAMP/PKA modulation; in fact, the AC activator forskolin increased its expression (43). Similarly, also dbcAMP, a lipophilic analog of cAMP which specifically activates PKA, significantly up-regulated the ABCB1 gene product, with an effect that was prevented by haemocyte pre-treatment with the PKA inhibitor H89. In addition, mRNA levels for the mussel 5-HTR1 were readily increased in mussel hemocytes following treatment with 5-HT (44). Accordingly, the 5-HT1 receptor recently identified in oysters was highly expressed in digestive gland and hemocytes, and in mammalian cells transfected with the Cg5-HTR-1, exposure to 5-HT caused inhibition of AC and reduction in cAMP levels (34).

The classification of 5-HTRs of different bivalve species on a molecular basis (**Figure 1**), confirms that bivalve molluscs possess three 5-HTRs, one 5-HTR7 and two 5-HTR1. Relative expression of different 5-HT receptors in different tissues, and activation of the corresponding signaling pathways, would therefore be responsible for the different modulatory effects of serotonin on multiple physiological functions.

## The Serotonergic System as a Target for Aquatic Contaminants in Bivalves: The Example of Antidepressants

Different CECs have been shown to affect serotonergic components in bivalves. In the freshwater mussel *Elliptio complanata*, exposure to estrogenic chemicals present in municipal effluents affected 5-HT levels (45). In *M. galloprovincialis*, 5-HTR1 expression was modulated in mantle, digestive gland, gills and hemocytes by *in vivo* exposure to estrogenic compounds (46) and hexavalent chromium (47, 48). Alterations in serotoninergic systems were observed in gills of mussels caged at a highly polluted area (49).

However, when addressing the issue of neuroendocrine disruption by environmental contaminants, the release of pharmaceutical residues in aquatic ecosystems represents the main cause of concern (50). This particularly applies to antidepressants, among the most commonly detected human pharmaceuticals in the aquatic environment, whose mode of action is modulation of conserved neurotransmitters in ecologically relevant invertebrate groups (molluscs and crustaceans), in particular serotonin (reviewed in 51).

The most widely prescribed antidepressants worldwide are fluoxetine and sertraline, acting as selective serotonin reuptake inhibitors (SSRIs), and venlafaxine and duloxetine, acting as serotonin-norepinephrine re-uptake inhibitors (SNRIs); these pharmaceuticals are in fact included within the 40 top prescribed pharmaceuticals (https://clincalc.com/DrugStats/Top200Drugs.aspx accessed on Jan 16 2022). The inhibition of SERT substantially increases the permanence of 5-HT in the synaptic cleft, prolonging its action.

Waterborne antidepressants are accumulated in bivalves, in the field as well as in laboratory conditions, and are at least in part metabolised. Nevertheless, the continuous discharge of pharmaceuticals in the aquatic environment makes them pseudo-persistent, and animals can be exposed for their whole life cycle. Occurrence (52) and effects (53) of antidepressants have been studied in aquatic animals, and striking effects were reported on marine invertebrate species (see 54 for an overview). Fluoxetine, widely detected in the aquatic environment, is by far the antidepressant most used in biological experiments. More recently, prescriptions of venlafaxine increased significantly, so that it became one of most frequently found antidepressant in effluents and surface waters, also due to its exceptionally low removal efficiency in waste water treatment plants (55). In general, antidepressant concentrations range from µg/L in inland waters to ng/L in marine waters, with values greater than 500 ng/L reported in urban estuaries (see for review 54, 56, 57).

In *M. galloprovincialis* venlafaxine accumulation reached an average tissue concentration of 2.146 ± 156 ng/g dry weight (dw) after 7 days of exposure to a nominal concentration of 10 µg/L. The bioconcentration factor (BCF) was 265 mL/g dw. After 7 days of depuration, a rapid decrease in the tissue concentration to 21 ± 1.0 ng/g dw was observed (58). After exposure to different concentrations of venlafaxine (1, 10 and 100 µg/L), metabolites were found in mussel tissues, namely N-desmethylvenlafaxine and O-desmethylvenlafaxine; also N,O-didesmethylvenlafaxine and N,N-didesmethylvenlafaxine were detected after exposure to the highest concentrations (58). These data indicate that *M. galloprovincialis* takes up venlafaxine and actively metabolises the drug. Among metabolites, at least N-desmethylvenlafaxine was reported to contribute to the therapeutic effects of venlafaxine in humans (58). The ability of mussels to uptake and metabolize venlafaxine has been confirmed by targeted and non-targeted approaches using liquid chromatography combined with high-resolution mass spectrometry to screen for expected metabolites based on the literature on aquatic species, and for metabolites not previously documented (59). Sertraline was found in *M. galloprovincialis* collected along the Catalan sea coast, Spain, at 1.5 ng/g wet weight (60). Similar values are reported in benthic mussels (*Geukensia demissa*) collected from the San Francisco Bay (61). Higher levels were detected in freshwater mussels (*Lasmigona costata*) collected in the field (29-77 ng/g ww) or caged in Grand River, Ontario, Canada (6-26 ng/g ww) (62). About 70% of 1225 mussels sampled along the Portuguese Atlantic coast appeared contaminated with at least one, and up to 4 antidepressants, with a cumulative content of SSRIs and their metabolites reaching 33.93 ng/g dw, and a mean level of about 15 ng/g dw. The main metabolite from fluoxetine, i.e. norfluoxetine, was the most recurring compound, which also showed the highest mean concentration of 13.52 ng/g (63).

Several effects of antidepressants were shown in bivalves. Fluoxetine mimics the action of a continuous exposure to increased extracellular levels of 5-HT (64). In *M. galloprovincialis in vivo* exposure to fluoxetine (0.3 ng/L) provoked a significant decrease in cAMP levels and PKA phosphorylation in the digestive gland, consistent with the increased serotonin levels in the presence of fluoxetine and binding to 5-HTR1 (43, 65). Interestingly, mRNA levels for the mussel 5-HTR1 were strongly increased by exposure to 0.03-3 ng/L fluoxetine, and recovered at basal levels at higher concentrations. Fluoxetine also decreased the level of ABCB1 transcripts, thus the expression of P-glycoprotein in mussel tissues and hemocytes (43, 66). P-glycoprotein is a key component of the multixenobiotic resistance (MXR) mechanism of cytoprotection, which naturally prevents the cellular accumulation of harmful xenobiotics by active extrusion. This mechanism is considered crucial for aquatic organisms to cope with polluted environments (67). Given this function, the inhibition of cAMP/PKA pathway and P-glycoprotein expression caused by fluoxetine at environmental concentrations may seriously affect the ability of animals to elaborate strategies of defense against chemical exposure. Fluoxetine also reduced lysosomal membrane stability (LMS) in mussel hemocytes from concentrations as low as 0.03 ng/L (65). LMS was the most sensitive biomarker; however, also neutral lipid accumulation, lipid peroxidation, lipofuscin and activity of acetylcholine esterase (AChE) and antioxidant enzymes were affected in the range 0.03-30 ng/L (65).

In the tropical brown mussel *Perna perna* exposed to fluoxetine, cholinesterase inhibition occurred at concentrations of 3 and 30 ng/L (48 h) and 30 and 300 ng/L (96 h) (68). GST activity was significantly increased after 48 h of exposure to 30 ng/L, and 96 h of exposure to 3 and 30 ng/L, respectively. DNA damage was also detected in the digestive gland after 48 h of exposure to 30 ng/L. Significant effects of fluoxetine on lysosomal membrane stability was observed from the lowest concentration of the compound (3 ng/L) after exposure to both 48 and 96 h. In *M. californianus*, long-term exposure (107 days) to environmental concentrations of fluoxetine (0.3-300 ng/L) reduced algal clearance in mussels, shell growth (length and biomass) and gonadosomatic index (69), this supporting the chronic effects at low doses. Exposure of the freshwater mussel *D. polymorpha* to fluoxetine for 6 days at environmentally relevant concentrations (20 and 200 ng/L), induced spawning, alteration of oocyte and sperm densities and of endogenous levels of esterified estradiol (70). In the same species, spawning was significantly induced within minutes of addition of fluoxetine or fluvoxamine at concentrations of 300 ng/L and 430 ng/L, respectively (71). Effects of fluoxetine were also found in the unionid mussel *Lampsilis fasciola*, characterized by a complex life cycle, where their glochidia larvae parasitize a vertebrate host, usually a fish (72, 73). In this species, the likelihood of metamorphosis of glochidia to the juvenile stage in the fish host was significantly enhanced after a 24 h exposure to 1-100 µg/L fluoxetine (72). In adults, exposure to fluoxetine for 28 days to 0.37 and 29.3 µg/L, or for 67 days at 2.5 and 22.3 µg/L, induced significant effects of different behavioral endpoints involving the mantle and foot (72, 73). Overall, given the unique life cycle of unionids, environmental exposure to fluoxetine may affect their interaction with the host, their susceptibility to predation and needs for energy substrates, this exacerbating the issue of conservation of this endangered species in North America (72).

Overall, the serotonergic system of bivalve molluscs can be affected by exposure to antidepressants also at environmental concentrations, i.e. in the low ng/L range, as firstly reported by Fong (74). Even more intriguing are the observations that the effects of antidepressants can be observed in very short periods of time (71, 74).

Effects at low concentrations, non-monotonic dose-responses and quick effects observed in invertebrates in comparison with those observed in vertebrates raised some debate in the scientific community (75, 76). Different studies reported that invertebrate species are extremely sensitive to fluoxetine (43, 65, 68–74, 77, 78). The key question was whether chronic exposure to the low concentrations expected for pharmaceuticals in the environment had adverse effects on aquatic organisms.

Sumpter and Margiotta-Casaluci (75), on the basis of the pharmacodynamics of fluoxetine in human patients (79), calculated that the therapeutic concentration of fluoxetine is in the range of 50-500 ng/mL. Therefore, to account for the effects observed at low doses in some invertebrates, fluoxetine would be surprisingly more potent in invertebrates than humans. In their commentary, it was recommended to collect more data at the highest standards possible, since these results, if confirmed, would have major regulatory implications (75). Fabbri and Franzellitti (76) replied highlighting that pharmaceuticals are bioactive compounds specifically designed to act at low concentrations when target organisms possess specific receptors; further, pharmaceuticals with non-steroid mode of action have the potential to affect wildlife homeostasis and reproduction at low doses; moreover, since standard ecotoxicity tests are inadequate to reveal subtle effects of neurohormone-like effects, new methods need to be developed (76). Within the same debate, Di Poi et al. (80), underlined that the severity of the effects could be species and age-dependent, and related to the mode and duration of exposure. In particular, cognitive abilities were impaired at 1 ng/L 5-HT in young cuttlefish (*S. officinalis*) exposed from 15-day pre-hatching to 30-day post-hatching, which is the most critical period for brain processes development in the cuttlefish (78). More recent data have been reported in *S. officinalis* juveniles exposed for 30 days to 5 ng/L fluoxetine or to a mixture of fluoxetine/venlafaxine at either 2.5 ng/L or 5 ng/L of each substance and examined weekly (81). The occurrence of sand-digging behaviour decreased in response to the mixture fluoxetine/venlafaxine at the lowest concentration (2.5 ng/L each). Using the mixture at 5 ng/L each, body covering after digging became more effective in juvenile shore crabs *Carcinus maenas* (82).

Some hypotheses have been proposed to justify the striking temporal or low-dose induced effects of antidepressants in aquatic invertebrates. Because bivalves filter high amounts of water, the gills and the entire mantle cavity containing the gonads, foot, digestive gland, and adductor muscles, would be directly exposed to the water and interaction of waterborne chemicals with external receptors would be possible (71). Similarly, also crabs, crayfish, shrimps, other crustaceans and worms may be largely exposed to waterborne antidepressants through the gills, the body surface and *via* ingestion (71).

Fong et al. (21) postulated that SSRIs could act not only as 5-HT reuptake inhibitors but also as agonists of the 5-HT postsynaptic receptors. This hypothesis is corroborated by studies on the nematode *Caenorhabditis elegans*, showing that fluoxetine binds directly to G-protein coupled receptors (83). Moreover, serotonin-dependent and -independent responses of fluoxetine were reported in *C. elegans* SERT-deficient mutants (84) and in 5-HT–deficient mutants (85).

## The Serotonergic System of Early Bivalve Larvae as a Target for Aquatic Contaminants: Potential Implication of Exposure to EDCs and NEDs

When considering the diversity of invertebrate neuroendocrine systems, it has been pointed out that early developmental stages represent a good experimental model that can provide significant information on critical steps in development of neurosecretory cells (14, 15). Consequently, studies on early larval neurodevelopment, together with the characterisation of the possible effects and mechanisms of action of NEDs, could be essential in the understanding of the true “endocrine” impact of EDCs on invertebrates (8, 86). Ford and Fong (71) underlined that some aquatic species are likely to be exposed either continuously or sporadically throughout their life histories, especially during critical life stages; in this light, it will be important to evaluate the long-term impacts of different EDCs and NEDs on neural development.

Marine invertebrate larvae and early life stages have long been used as experimental models in various disciplines and not exclusively related to environmental issues (87, 88). Nowadays, knowledge on larval development of a number of marine invertebrates from distinct phyla is also exploited by standard embryotoxicity tests (www.iso.org, www.astm.org). Actually, marine invertebrate larvae meet almost all the characteristics of good experimental model organisms: they are inexpensive, easy to obtain from adult specimens, they develop fast and are easy to grow in laboratory conditions; moreover, their utilization for research and regulatory purposes is not affected by ethical restrictions, and the collection of ripening adult specimens from the environment does not generally impose risks to deplete natural populations (89–91). Calcifying larvae of marine invertebrates in particular have been shown to be particularly sensitive to environmental pollutants and stressors (92, 93). Finally, the fast larval morphogenesis and early developmental transitions of marine invertebrates could serve as potent endpoints of adverse effects of EDCs in the context of application of Adverse Outcome Pathways (AOPs) and establish neuro-endocrine related biomarkers specific to invertebrates (94, 95). However, little information is available on the effects and mechanisms of action of EDCs and NEDs on early development of most marine invertebrates.

An interesting example is that of the effect of subchronic exposure to fluoxetine (1 and 10 μg/L) during the last 15 days of embryonic development of *S. officinalis* (96). In particular, fluoxetine modulated dopaminergic but not serotonergic neurotransmission, and decreased cell proliferation in key brain structures for cognitive and visual processing. It was postulated that “a long-term disturbance of the dopaminergic function during critical periods of development may lead to a disruption of the synaptic function and may thereby drive abnormal neurodevelopmental processes, impair complex behaviors of the juvenile cuttlefish and thus lead to a decrease in their survival” (96).

Knowledge on larval development of different bivalve species, mussels and oysters in particular, has greatly increased in the last few years, also thanks to the development of microscopical techniques and –omics technologies that have been mainly applied to investigate key physiological processes, such as biomineralization and immune response (97–100). In the oyster *C. gigas* several antidepressants, the SSRIs fluoxetine and sertraline, tricyclic antidepressants (TCAs) clomipramine and amitriptyline, the SNRI duloxetine, were tested in a wide concentration range on D-shaped larvae at 36 hours post fertilization (hpf) (101). For all chemicals, the EC_50_ values were within the same range of concentrations (67 to 192 μg/L) and higher than environmental levels. However, the mechanisms of action were not investigated.

In contrast, several potential EDCs and NEDs have been shown to affect early larval development (48 h) in the mussel *M. galloprovincialis* at environmental concentrations (102–104). With regards to the possible molecular mechanisms involved, it was demonstrated that both the natural estrogen 17β-estradiol and the xenoestrogen bisphenol A (BPA) modulated the transcriptional profiles of different genes, in particular of those involved in shell formation and neuroendocrine signalling, including the 5-HTR1 (105). Other widespread pharmaceuticals (diclofenac, carbamazepine, propranolol) also affected the transcription of 5-HTR1 and of genes involved shell biogenesis at environmental concentrations (104, 106). Although the extent and effects (up/downregulation) on 5-HTR1 expression were different depending on the compound, concentration, and larval stage (24 or 48 hpf), these data pointed at serotonin signalling as a common target for a variety of EDCs and NEDs in mussel early development. Moreover, the results strongly suggested a link between disruption of the processes involved in early neurogenesis and shell biogenesis.

**Figure 2:**
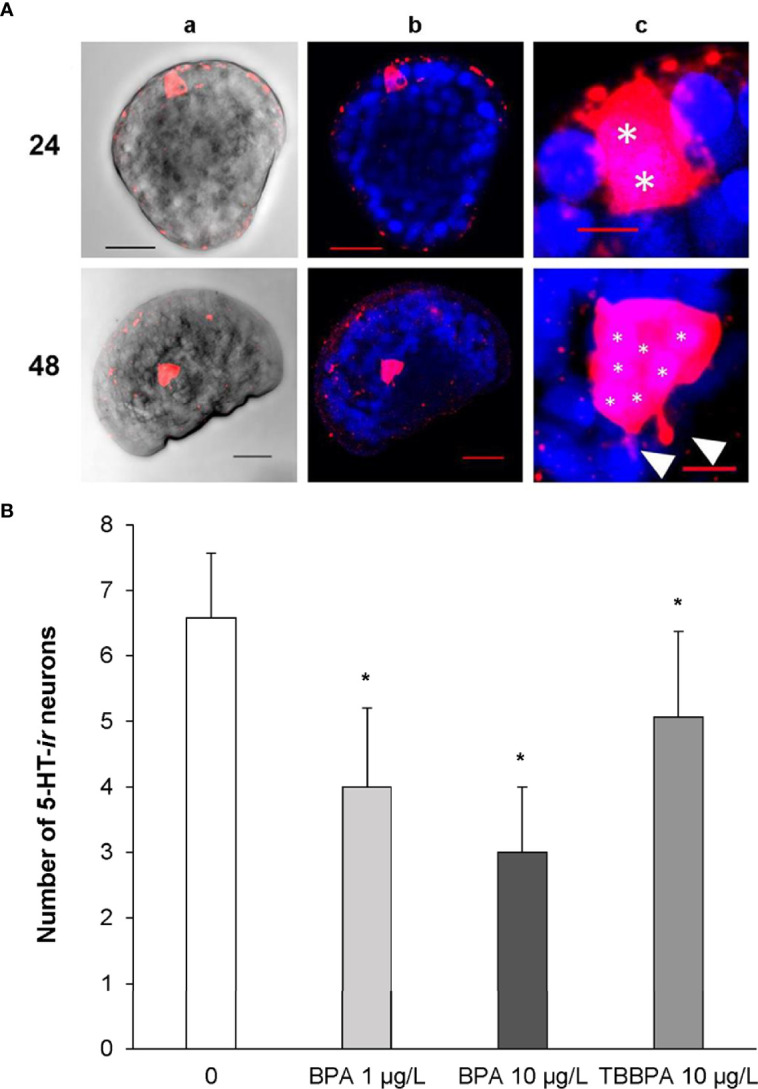
Serotonin immunoreactive cells (5-HT-*ir*) in early larval stages of *M. galloprovincialis.* **(A)** Confocal images of 5-HT-*ir* neurons at 24 and 48 hpf. 5-HT-*ir* cells are shown in Red/Pink (Ex/Em: 590/617 nm), Hoechst stained nuclei are shown in blue (Ex/Em: 358/461 nm). a) brightfield images, scale bar: 20 µm; b) 5-HT and Hoechst merged channels, scale bar: 20 µm; c) 4-5 X magnified images of column; white asterisks indicate the nuclei of 5-HT-*ir* cells (*) and white arrowheads emerging neurites, respectively, scale bar: 5 µm. **(B)** Effect of BPA (1 and 10 µg/L) and TBBPA (10 µg/L) on development of 5-HT-*ir* cells in mussel larvae at 48 hpf. The number of 5-HT-*ir* cells was quantified in at least 12 larvae from 4 different parental pairs (N=4). Data are reported as mean ± SD, indicating significant differences between control and BPA and TBBPA-exposed samples. *p < 0.01 (Mann-Whitney U test). For methods, see Miglioli et al. (108).

Therefore, development of serotonergic components were investigated in more detail between 24 and 48 hpf, when the key processes leading to the formation of the first larval shell occur (107), in larvae exposed to BPA and to its brominated derivative, the flame retardant tetrabromobisphenol A (TBBPA) (108, 109). Basal expression of 5-HTRs indicated a strong upregulation in early larvae (24 hpf) with respect to eggs, followed by a decrease at 28 and 32 hpf and a further increase at 48 hpf (**Figure S1**). Accordingly, the earliest 5-HT immunoreactive (5-HT-*ir*) neurons were detected at 24 hpf (trochophora) in the region of the developing apical organ (AO), the most conserved larval sensory structure, which later becomes a part of the emerging cerebral ganglion; the number of 5-HT-*ir* cells progressively increased up to seven at 48 hpf, with the appearance of tiny neurites (see representative images in **Figure 2A** and 108).

Exposure to BPA at environmental concentrations (1 µg/L) induced significant downregulation of 5-HTR only at 48 hpf; however, BPA affected the development of 5-HT-*ir* neurons, with a decrease in their number from 28 hpf (108). At 48 hpf, the extent of reduction in cell number was associated with different degrees of developmental delay (early veligers and larvae withheld at the trocophora stage) (108). Higher concentrations (10 µg/L) resulted in even stronger reductions in the number of 5-HT-*ir* neurons (**Figure 2B**). Irrespective of the concentration, the most evident effects of BPA were detected at 48 hpf, when both the number of 5-HT-*ir* cells and the amount of 5-HTR transcripts were reduced. In contrast, TBBPA did not affect mRNA levels for 5-HTR at any time pf (109). However, TBBPA, although at higher concentrations (10 μg/L) induced a decrease in the number of 5-HT-*ir* neurons at all times pf, with a significant decrease with respect to controls at 48 hpf (**Figure 2B** and 109) (**Figure 2B**). Moreover, TBBPA altered the expression pattern of components of dopamine metabolism and signalling (tyrosine hydroxylase, dopamine-β-hydroxylase and dopamine receptor 1), and affected development of GABAergic neurons (109).

Overall, the results demonstrate that in mussel early larvae exposure to known EDCs interfere with neurogenesis of the serotoninergic system. The effects were associated with altered shells and expression of key genes involved in shell biogenesis, tyrosinase and chitinase in particular, resulting in altered larval phenotypes (107–109). These observations also support the hypothesis that first shell formation, a key step in early bivalve development, is modulated by monoamine neurotransmitters 5-HT and dopamine (DA) through TGF-β Smad mediated pathways that trigger the expression of tyrosinase to form initial shell (110).

Overall, increasing knowledge on development of neuroendocrine systems in early larval stages of bivalves and how these processes can be affected by potential EDCs or NEDCs will help identifying the mode of action (MOA) of these chemicals in a relevant group of marine invertebrates.

## Conclusions and Perspectives

The serotonergic system represents one of the most conserved neuroendocrine pathway in invertebrates. In bivalve molluscs, widespread in all aquatic environments that are subjected to contamination by a variety of CECs, 5-HT modulates multiple physiological functions in different cells and tissues. Functional and molecular evidence support the presence of 5-HT1 and 5-HT7 receptors and related signaling pathways in most common bivalve species. Available information on the effects and mechanisms of action of different CECs (EDCs and antidepressants in particular), underline how the serotonergic system can represent a sensitive target for different neuroendocrine disruptors, in both larvae and adults.

**Figure 3:**
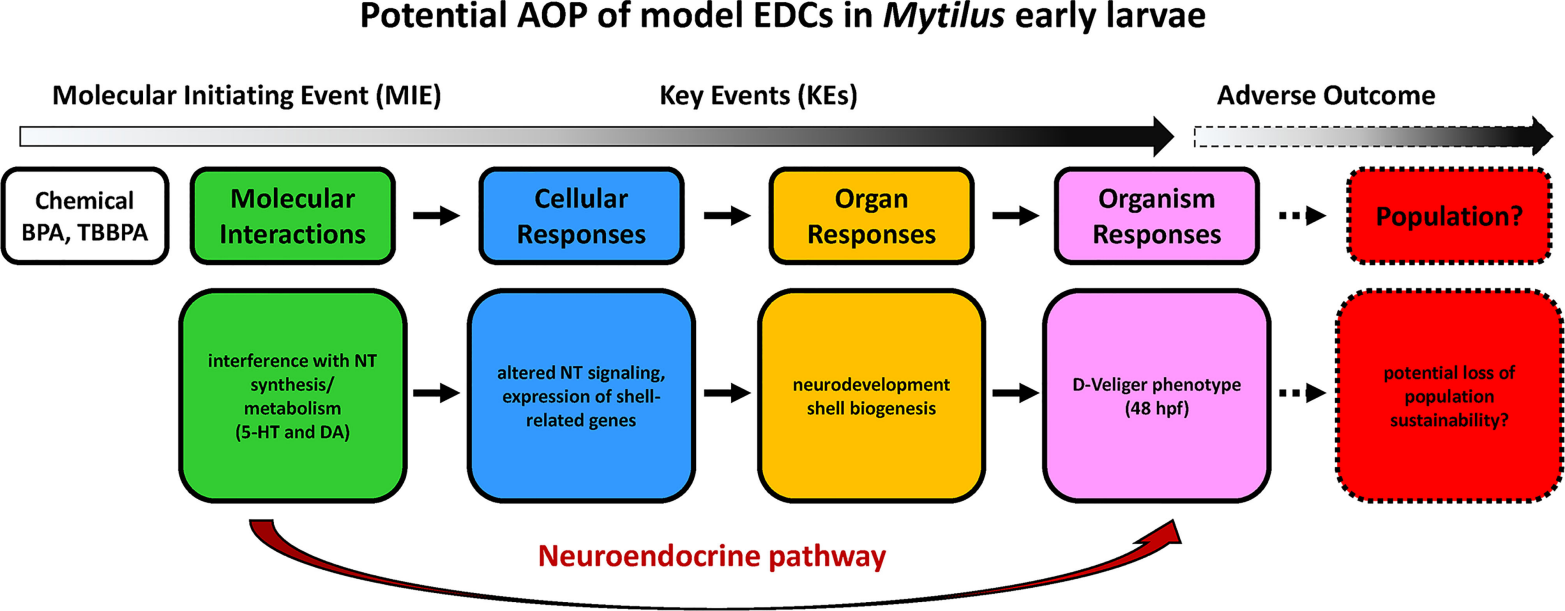
An AOP outlining the impacts of model EDCs on *Mytilus* early larval development, based on data on perturbation of neuroendocrine pathways induced by BPA and TBBPA (108, 109). NT, neurotrasmitters. Words in italics suggest possible effects for which empirical data does not yet exist.

With regards to early larval stages, although the complex signaling pathways linking neurodevelopment with shell biogenesis need to be fully elucidated, the results obtained in *M. galloprovincialis* represent, to our knowledge, the most extensive data so far on the effects and mechanisms of action of CECs in bivalves. The results obtained for model EDCs such as BPA and TBBPA indicate that these compounds can act as neurodevelopmental disruptors in mussel early larvae. These data can contribute building a first AOP in early bivalve larvae involving the serotonergic system, where molecular initiating events (MIE) caused by the initial exposure (i.e. alteration of synthesis or metabolism of 5-HT and other monoamines like DA), can lead to a series of key events (KE) that, from disruption of neurotransmitter signalling, affect not only neurodevelopment but also the processes involved in shell biogenesis (expression of genes involved in organic matrix deposition and consequent calcification). The overall effects can be identified at the organism level as altered larval phenotypes (malformed, delayed, arrested larvae), that may in turn result in potential loss of population sustainability (**Figure 3**).

**Figure 4:**
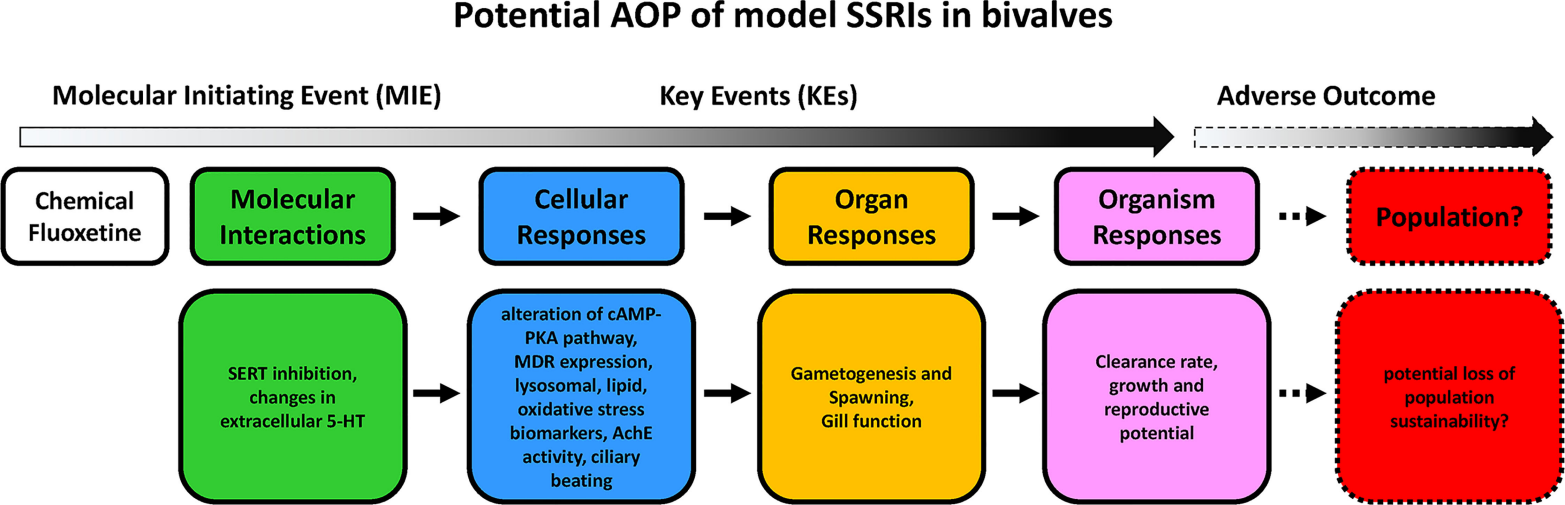
An AOP outlining the impacts of model SSRI on bivalves, based on data obtained with environmental concentrations of fluoxetine quoted in the reference list. Words in italics suggest possible effects for which empirical data does not yet exist.

In adult bivalves, building AOPs for potential disruptors of the serotonergic system still appears a more complex task. Although a number of effects of exposure to environmental levels of model SRRIs have been described, in particular fluoxetine, at the cellular, tissue and whole organism level, less information is available on the molecular interactions. Modulation of main serotonin-related intracellular signaling pathways, cAMP-PKA in particular, seems to represent the most common effect at the molecular/cellular level. Due to the pleiotropic roles of 5-HT, this can result in multiple effects in different tissues and at the whole organism level, and contribute to predict the overall impact at population level. A tentative AOP for fluoxetine based on available data obtained at environmental concentrations in bivalves is reported in **Figure 4**.

Invertebrates obviously possess conserved components of the neuroendocrine system other than serotonin e.g. catecholaminergic, cholinergic, enkephalinergic, dopaminergic, gamma-aminobutyric acid-ergic, and neuropeptide systems (13, 111), whose pathways can be strictly interconnected, as suggested by data obtained in cuttlefish embryos with fluoxetine (96) and in mussel larvae with TBBPA (109). In addition, it must be considered that adrenergic signaling seems absent in invertebrates, and analogous functions are performed by the biogenic amines octopamine and its precursor tyramine, that act *via* related families of G-protein-coupled receptors and are regarded as the invertebrate equivalents of vertebrate norepinephrine (reviewed in 112). However, the effects of potential neuroendocrine disruptors on octopamine and tyramine-mediated pathways have been seldom considered so far. Back in the eighties serotonin and octopamine were reported to induce opposite effects on aggressive behaviour in crustaceans: in particular, injection of serotonin produced postures resembling those seen in dominant animals during/after agonistic encounters, while octopamine produced postures resembling those seen in subordinate animals (see for review 113). Infusion of Prozac, i.e. fluoxetine, in subordinate animals led to a significant reduction in the 5HT-mediated increases in fight duration (114), suggesting that exposure to fluoxetine may impair social equilibrium. Data on behavioural endpoints, if supported by molecular evidence, may greatly help building AOPs for different compounds.

Overall, basic research on neuroendocrine signaling is still needed to evaluate the potential impact of potential neuroendocrine disruptors in key invertebrate groups. In this light, Ford and Fong (71) underlined the importance of publishing negative and/or non significant results to aid risk assessment of antidepressants as well as of EDCs. This publication bias, that is among the common issues that limit the policy impact of environmental science research (115) is of particular relevance in invertebrate studies with CECs, given the number of different phyla and species, the variability between the type of chemical, model species, experimental designs and endpoints evaluated in different studies. Sharing this type of results will not only help the scientific community filling research gaps, but also provide both positive and negative findings that will help policy makers to make well-informed decisions.

## Author Contributions

LC and EF conceived the manuscript. AM and TB performed experiments. LC, EF, AM, and TB writing and editing. All authors have read and agreed to the published version of the manuscript.

## Funding

This work was supported by MIUR-RFO 2019 funds to LC and EF.

## Conflict of Interest

The authors declare that the research was conducted in the absence of any commercial or financial relationships that could be construed as a potential conflict of interest.

## Publisher’s Note

All claims expressed in this article are solely those of the authors and do not necessarily represent those of their affiliated organizations, or those of the publisher, the editors and the reviewers. Any product that may be evaluated in this article, or claim that may be made by its manufacturer, is not guaranteed or endorsed by the publisher.
