# Supplementary material for: Physiological Roles of Serotonin in Bivalves: Possible Interference by Environmental Chemicals Resulting in Neuroendocrine Disruption
Source: Front Endocrinol (Lausanne). 2022 Feb 25;13:792589. doi: 10.3389/fendo.2022.792589 (PMC8913902; doi:10.3389/fendo.2022.792589)
Supplement: Supplementary file 1 [file DataSheet_1.docx]

Supplementary Material to

# Canesi et al.: Physiological roles of serotonin in bivalves: possible interference by environmental chemicals resulting in neuroendocrine disruption

**
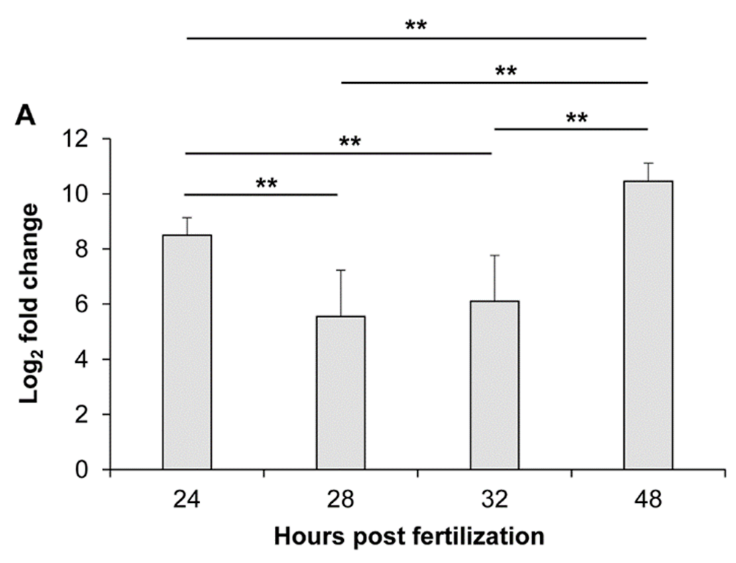
**

**Figure S1** – Basal expression of 5-HTR across early developmental stages of *M. galloprovincialis* evaluated by qPCR (for methods see Balbi et al., 2016). Data (mean ± SD), reported from the lowest to the highest level of expression, are shown as relative expression (log_2_-transformed fold changes) with respect to unfertilized eggs. *p < 0.05; **p < 0.01 (Mann-Whitney U test).

**Supplementary Table 1**

**
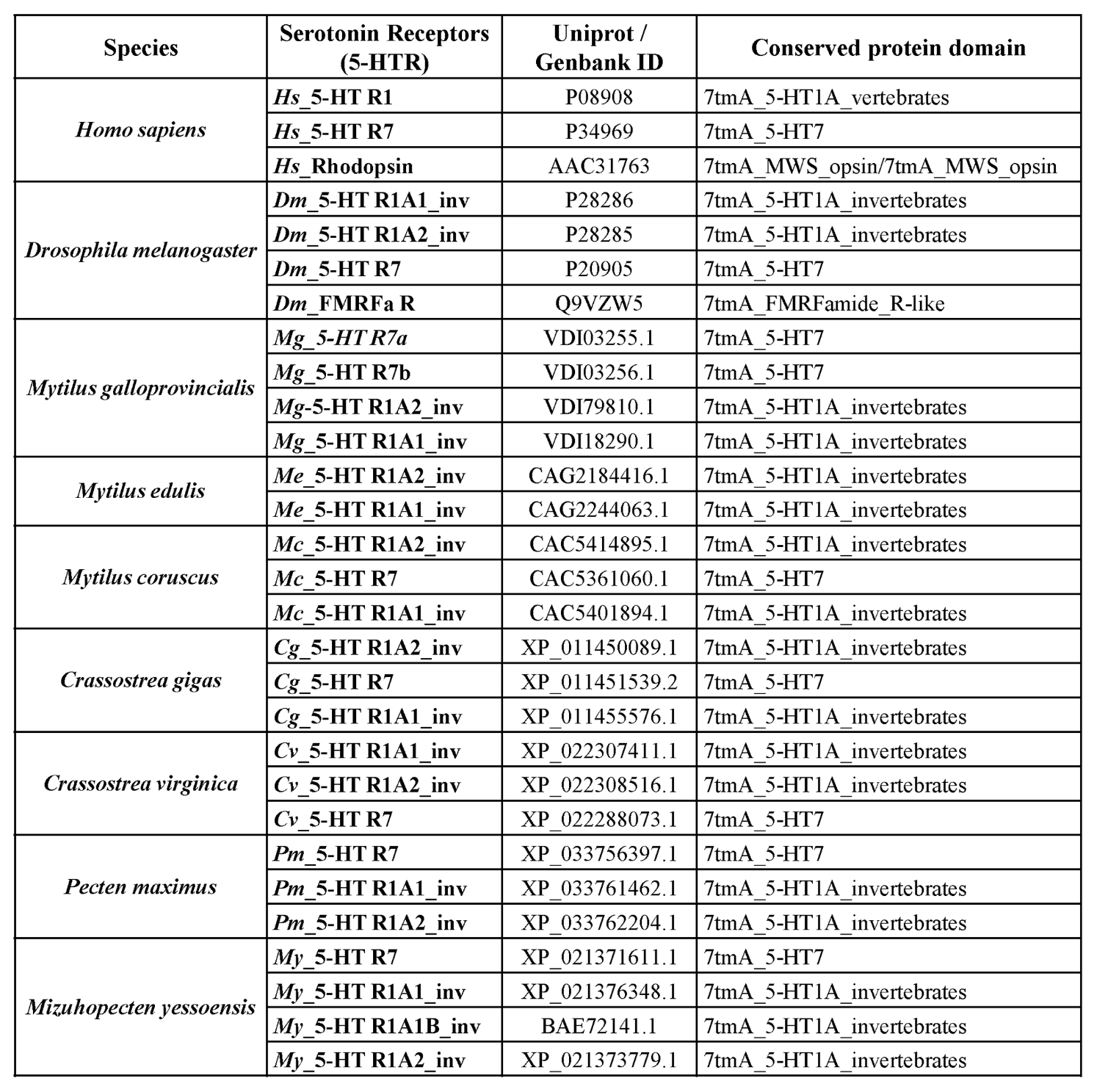
**

**Table S1** – 5-HT receptor sequences employed for the phylogenetic analysis from *Homo sapiens*, *Drosophila melanogaster, Mytilus galloprovincialis, Mytilus edulis, Mytilus coruscus, Crassostrea gigas, Crassostrea virginica, Pecten maximus* and *Mizuhopecten yessoensis.* The receptors were named and selected depending on the outputs of the conserved domain search by NCBI Conserved Domain database (Yang et al., 2020).

**References**

Balbi T, Franzellitti S, Fabbri R, Montagna M, Fabbri E, Canesi L. Impact of bisphenol A (BPA) on early embryo development in the marine mussel *Mytilus galloprovincialis*: Effects on gene transcription. Environ Pollut (2016) 218: 996-1004. doi: 10.1016/j.envpol.2016.08.050

Yang M, Derbyshire MK, Yamashita RA, Marchler-Bauer A. NCBI's conserved domain database and tools for protein domain analysis. Curr Protoc Bioinformatics (2020) 69: e90. doi: 10.1002/cpbi.90
